# Asbestos Ban in Italy: A Major Milestone, Not the Final Cut

**DOI:** 10.3390/ijerph14111379

**Published:** 2017-11-13

**Authors:** Daniela Marsili, Alessia Angelini, Caterina Bruno, Marisa Corfiati, Alessandro Marinaccio, Stefano Silvestri, Amerigo Zona, Pietro Comba

**Affiliations:** 1 Department of Environment and Health, Istituto Superiore di Sanità, 00161 Rome, Italy; caterina.bruno@iss.it (C.B.); amerigo.zona@iss.it (A.Z.); pietro.comba@iss.it (P.C.); 2 Institute for Study and Prevention of Cancer, 50139 Florence, Italy; a.angelini@ispo.toscana.it (A.A.); s.silvestri@ispo.toscana.it (S.S.); 3 Department of Occupational and Environmental Medicine, Epidemiology and Hygiene, Italian Workers Compensation Authority (INAIL), 00143 Rome, Italy; m.corfiati@libero.it (M.C.); a.marinaccio@inail.it (A.M.)

**Keywords:** asbestos, Italy, mesothelioma, asbestos-related disease, environmental cleanup, health surveillance, epidemiological monitoring, international scientific cooperation

## Abstract

**Background and history:** Italy was the main asbestos producer and one of the greatest consumers in 20th century Europe until the asbestos ban was introduced in 1992. Asbestos exposure affected the population in a wide range of working environments, namely mining and marketing of asbestos, asbestos cement production, shipyards and textile industries. This also determined a widespread environmental asbestos exposure affecting the surrounding communities. **Methods:** To investigate the drivers and difficulties of the process leading to the asbestos ban and its subsequent implementation, we focused on stakeholder involvement, environmental health policies, capacity building and communication. **Results:** In the past three decades, stakeholder involvement has been instrumental in advancing the industrial asbestos replacement process, prevention and remediation interventions. Furthermore, involvement also contributed to the integration of environmental and health policies at national, regional and local levels, including capacity building and communication. In a global public health perspective, international scientific cooperation has been established with countries using and producing asbestos. **Discussion and Conclusions:** Key factors and lessons learnt in Italy from both successful and ineffective asbestos policies are described to support the relevant stakeholders in countries still using asbestos contributing to the termination of its use.

## 1. Foreword

Asbestos production, trade and consumption currently concern a large part of the world’s population in a considerable number of countries. In the past two decades, the global asbestos production has decreased, but not fallen below approximately 2,000,000 tons per year [1,2], mined by multinational and domestic asbestos industries. The international trade of chrysotile asbestos fibres and asbestos containing products has not yet been regulated [3]. This is happening in spite of scientific evidence of asbestos carcinogenicity for humans demonstrated and evaluated by IARC Monographs for several decades [4,5,6]; recommendations and guidelines for the elimination of asbestos-related diseases have been issued by WHO and ILO [7,8].

Political decisions to ban asbestos use at national levels have to take multi-sectoral implications into account due to existing mutual influence between health, environmental, industrial and occupational policies. An effective public health approach thus requires a multi-sectoral political framework promoting sustainability of industrial manufacturing and consumption of asbestos substitutes as well as the reduction of hazardous chemical use, to defend public health and environmental protection, in agreement with the Sustainable Development Goals (SDGs) of the United Nations 2030 Development Agenda [9].

Thirty-seven out of the fifty-three countries belonging to the WHO European Region have adopted asbestos bans [10]. The WHO Regional Office for Europe has reiterated its recommendations to all countries of this region to adopt national plans for the elimination of asbestos-related diseases in collaboration with WHO and ILO [10].

As far as the European Union (EU) is concerned, the Commission Directive 1999/77/E.C. established an asbestos ban in the EU to be implemented by 2005 in all its then twenty-five Member States [11]. At that time, Italy was implementing its national law banning asbestos approved in 1992 [12], after having been one of the main producers and consumers in 20th century in Europe [1]. The implementation of the Italian law banning asbestos listed multi-sectoral interventions, increasing awareness of asbestos risks and impacts, and advancing the industrial asbestos replacement process. In the past three decades, preventive actions and remediation interventions have been increasing, including the involvement of the relevant stakeholders as described below. Integration of environmental and health policies at national, regional and local levels has so far been incomplete, as a result of a wide range of critical situations, as discussed later.

From a global public health perspective, for the past decade, the Italian scientific cooperation has been instrumental in preventing asbestos-related diseases in countries still using asbestos.

The purpose of the present paper is to address the process that led to the enforcement of the asbestos ban in Italy and the subsequent actions including asbestos removal, environmental cleanup, waste management, epidemiological and health surveillance and communication. Emphasis in the paper will be given to the need to implement the law banning asbestos use, which would otherwise be ineffective. Moreover, the lessons learnt in Italy in preventing asbestos-related diseases are also described to support the relevant stakeholders in countries still using asbestos to terminate its use, in the framework of international cooperation.

## 2. Asbestos in Italy and the Steps Leading to the Law Banning Asbestos

In Italy, asbestos exposure affected people employed in a wide range of working environments, namely mining and marketing, asbestos cement production, shipyards and textile industry. The situation also determined a broad environmental asbestos exposure affecting surrounding communities [13].

Asbestos mining was an important industrial activity in Northern Italy for decades. Specifically, the Balangero quarry in the Piedmont Region, which was the largest European chrysotile quarry, opened in 1917, and finally closed in 1985 [14]. The then emerging Eternit multinational company started producing asbestos cement in Italy in 1907, establishing the large industrial plant in the city of Casale Monferrato (Piedmont Region). Starting from the 1930s, other industrial asbestos cement plants operated in the cities of Broni (Lombardy Region) and Bari (Apulia Region) for decades. Excesses in the incidence of mesothelioma are still recorded in these areas [15]. All the above locations are listed as Italian National Priority Contaminated Sites for environmental remediation [16]. A higher than expected incidence in mesotheliomas and mortality have also been observed in areas with the naval shipyards, and other industrial activities where asbestos fibers and asbestos-containing products were in large use, such as petrochemical and steel plants [17,18]. The annual burden of asbestos-related diseases due to occupational, environmental and domestic exposures in Italy currently includes 400 new cases of asbestosis [19] and 1500 new cases of mesothelioma [20]. Estimates of the annual number of asbestos related neoplasms may be found in references [21,22].

According to Vigliani [23], the involvement of Italian researchers in investigating asbestos risks and health impact dates back to 1908–1910 with two studies describing the incidence of tuberculosis in asbestos industry workers and a lethal case of lung asbestosis complicated by tuberculosis. In the late 1940s, studies by Vigliani & Mottura [24] subsequently presented at the Symposium of the New York Academy of Sciences “Biological aspects of asbestos” held in 1964, indicated the carcinogenicity of asbestos fibers. In the following years, several Italian studies provided evidence of the health impact of asbestos exposures concerning traditional occupational settings such as asbestos cement factories [25], shipyards [26,27], and, unsuspected at that time, industrial sectors like railway carriages construction and repair [28], and non-asbestos textile industry where rags were packed in previously asbestos-containing jute bags [29].

The commitment of the Italian National Institute of Health (Istituto Superiore di Sanità—ISS) on asbestos started in 1980 through the establishment of a multidisciplinary research group to undertake epidemiological, environmental and electron microscopy studies on asbestos. Two research instruments were used to study the health impact of asbestos: (i) electron microscope determination of fibres in both environmental and biological matrices, and (ii) geographic studies of malignant pleural neoplasms: mapping mortality in the 20 Italian Regions, 100 Provinces and 8000 Municipalities [30].

ISS training activities for health and environmental professionals were also undertaken in cooperation with the National Institute for Occupational Safety and Prevention (Istituto Superiore per la Prevenzione e la Sicurezza del Lavoro—ISPESL), which was subsequently merged with the Italian Workers Compensation Agency (Istituto Nazionale Assicurazioni contro gli Infortuni sul Lavoro—INAIL).

Regional Environmental Authorities, operating in the areas of the country with a massive presence of asbestos industrial activities, developed skills for asbestos sampling, and subsequent analytical determinations. Likewise, Regional and Local Health Authorities operating in the same areas involved in the recognition of asbestos-related diseases, including diagnostic procedures and epidemiological investigations. Cooperation between national and regional/local institutions as well as with trade unions grew in the 1980s to support the implementation of the national asbestos ban.

The work of the Italian Parliament approving the asbestos ban lasted four years and the Italian law banning asbestos came into force in 1992—Law 257/92 (Legge 27 marzo 1992, n. 257, “Norme relative alla cessazione dell’impiego dell’amianto”) [12]. This law was preceded by Decree n. 277, 15/08/1991 implementing the European Directives 80/1107/EEC, n. 82/605/EEC, n. 83/447/EEC, n. 86/188/EEC and n. 88/642/EEC on the protection of workers from the risks arising from exposure to chemical, physical and biological agents at work [31]. A pilot initiative for banning asbestos in a local context was adopted by the Mayor of Casale Monferrato in 1987 in light of the dramatic situation of asbestos-related diseases in that town where, as previously mentioned, the main Italian Eternit plant was located.

The most important provisions of Law 257/92, prohibiting extraction, import, export, marketing and production of asbestos or asbestos containing products, included a consistent set of definitions and aims, criteria and methods for analytical controls, guidelines for governmental activities, procedures for managing asbestos-containing waste, plans for environmental clean-up, social security intervention to support former asbestos workers, and funding.

The long-lasting commitment of the Italian scientific community in investigating the health impact of asbestos supported the national policy-makers in adopting the law banning asbestos. After twenty-five years since the national asbestos ban, the Italian scientific asbestos community still has an ongoing commitment supporting institutional and social players in implementing the law, especially with respect to the prevention of asbestos-related diseases and environmental remediation in the most contaminated areas (see below).

## 3. Implementing Asbestos Policies after the Ban in Italy

The adoption of the law banning asbestos was a great achievement, as discussed in the previous sections. The law then required to be implemented and this process (still in progress) implied a set of actions that can be summarized as follows:

(1) *Public policies for asbestos substitutes*. During the 1980s, when the knowledge of the health risks caused by asbestos was spreading, some industries researched possible substitute materials to replace asbestos without compromising their technological functionality [32]. Since there was a risk that the new materials would have been mistakenly considered harmless just because they did not contain asbestos and were therefore used without any precaution, guidelines for their safe use were provided [33]. Asbestos substitutes were selected on the basis of the size of the fibres and their possible solubility in the respiratory system. It is well known that asbestos fibres fracture longitudinally, thus producing fibres with an even smaller diameter while keeping the length unaltered. Most of these materials were and are still made of man-made mineral fibres (MMMF) such as glasswool, rockwool, slagwool and ceramic fibres [34,35,36,37,38,39,40,41]. IARC published a monograph that classified MMMFs as possibly carcinogenic to humans, allocating them in group 2B [42], but in 2002 it only retained ceramic fibres in this class and moved the others into group 3, that is, “not classifiably as to carcinogenicity to human” [43]. The choice of a substitute material among MMMFs could be done taking the following into account: (i) filaments are preferable to wool, where technical requirements allow it, as their geometric diameter prevents them from being breathed in; (ii) glass materials are preferable because they are more soluble in biological liquids, even more so if the material to be used must necessarily be mineral wool. Since the ban, the products that employed the largest quantities of the mineral (asbestos-cement and friction materials) are now manufactured with cellulose mixed up with cement, organic artificial fibres (kevlar) and inorganic (MMMF) mixed up with phenolic resins. From a strictly economical point of view, substitutes are on average more expensive than asbestos. However, it should be said that if the manufacturing of asbestos products had always been performed with dust control systems, its cost would never have been competitive. Asbestos cannot be replaced by just one single material. Every application generally requires a specific material. Most substitutes are not classified as carcinogenetic or queried as carcinogenetic except for ceramic fibres. From a technological point of view, replacement of asbestos with other materials has now been successful in 100% of cases.
(2) *National surveillance system of mesothelioma incidence.* After the 1992 asbestos ban in Italy, there was a growing need for epidemiological surveillance of malignant mesothelioma incident cases, consistently with European Directive 83/477/CEE [44]. A permanent surveillance system of mesothelioma incidence was set up in 2002 by the National Register of Malignant Mesotheliomas (Registro Nazionale dei Mesoteliomi, ReNaM in Italian). The aims of ReNaM (set by law) are to identify cases, estimate mesothelioma incidence, assess asbestos exposure, particularly recognize unknown sources of contamination, and finally promote research projects. ReNaM has a regional organization: a Regional Operating Centre (Centri Operativi Regionali—COR, in Italian), which has been gradually established in all 20 Italian Regions. Each COR works by applying the national standardized methods, described in the national Guidelines [45]. By December 2016, ReNaM had gathered 27,035 MM cases, referring to the period of incidence between 1993 and 2015, and the modes of exposure to asbestos were investigated for 21,108 of them (78%). The epidemiological findings are described and discussed in details in ReNaM national reports [19]. The systematic collection of mesothelioma incident cases and of economic activities involved in asbestos exposure made it possible to have reliable information on the time-trend of the occurrence of disease [46], the territorial clusters of incident cases and their causes [17], the characteristics and extent, of environmental and familial modes of exposure, estimating the current weight of non-occupational exposure around 10% of cases [13]. Furthermore, the median period of latency and the determinants of survival rates have been estimated and discussed [47,48]. The aforementioned figures are important to correctly interpret the epidemic curve of mesothelioma in Italy, which so far has not yet decreased. The analysis of the occupations reported by the cases and their next of kin’s have contributed to improving compensation procedures [49]. Mesothelioma registration in Italy, besides providing a description of the occurrence of mesothelioma at a national level, has been crucial in interpreting localized clusters of mortality for malignant pleural cancer. This was the case in Biancavilla (Sicily) were mortality figures suggested the occurrence of an excess risk of pleural mesothelioma that was confirmed with respect to diagnostic procedures, risk estimates and causal agent, a previously unknown asbestiform fibre, subsequently named fluoro-edenite and classified by IARC as carcinogenic for humans [50,51,52,53]. The epidemiological systematic surveillance of asbestos-related diseases incidence, thus, is fundamental in preventing asbestos exposure, to support the efficiency of insurance and welfare systems and for producing epidemiological evidences.
(3) *Health surveillance for asbestos ex-exposed subjects and asbestos exposed workers.* After the national ban, health surveillance of workers still exposed to asbestos, i.e., those engaged in the maintenance, removal and disposal of asbestos containing materials, continued to be the employer’s responsibility and liability [31,54,55]. As regulated by Decree n. 257/2006 [56] and fine-tuned by art. 259 under the Decree n. 81/2008 [54] and subsequent updates, the asbestos exposed workers currently undergo a medical examination by an occupational health physician at least every three years. The examination includes lung function tests and, whenever required according to updated scientific knowledge, radiological or other diagnostic tests [54]. Exposure registries are also filled in at company/plant levels and transmitted to both the National Health System inspection authorities and, for epidemiologic purposes, to the Italian workers’ compensation authority (INAIL). The need to monitor the health status of workers after the cessation of asbestos exposure has been also considered in the Italian legislation since a medical examination at the end of the working life has been mandatory since the mid-1990s. In the course of the examination, the company doctor must inform workers about long-term possible adverse effects. Regional Governments are responsible for health facilities and different procedures were adopted by the various Italian regional administrations [57]. A great number of experiences of health care programs targeted to formerly exposed workers were conducted at regional or local levels, mainly involving the Prevention, Health and Safety at Work Services of the Local Health Units of the National Health System [57,58]. A joint WHO and Italian National Institute of Health research project “Cohort Studies in Areas of High Environmental Risk in Sicily” investigated, inter alia, former workers of an asbestos-cement plant located in San Filippo del Mela, near Messina (Sicily), in the Milazzo–Valle del Mela area. One of the project’s goals was to study the feasibility of a medical surveillance programme of former asbestos workers [59,60]. To standardize procedures and offer the same the health services and benefits to ex-exposed workers and patients, CCM (Center for Diseases Prevention and Control—National Ministry of Health), INAIL (National workers compensation authority) and the Conference of Regions and Autonomous Provinces drafted a National Protocol for health surveillance of asbestos ex-exposed workers [61]. The main features of this Protocol can be summarized as follows. It should be based on scientific evidence, and its contents should comply with declared aims. Criteria for inclusion, diseases to be identified, medical facilities involved, procedures for communication, and the role of general practitioners (GPs) should be clearly stated. Workers and employees involved should have the appropriate skills. Pneumologists, radiologists, pathologists, surgeons, cancer specialists (oncologists) and technical staff are essential. Liaison with specialized centers for the treatment of neoplasms, and in particular mesothelioma, will be very important. The minimum level of the health surveillance programme can be summed up as follows: (a) medical examination; (b) lung function tests (LFT such as flow/volume curve, Dl, co); (c) chest X-rays, CXR, performed and interpreted in accordance with ILO indications [62]; (d) smoking cessation; (e) planned procedures for handling cases requiring further diagnosis; (f) pneumococcal disease and influenza vaccinations [63]. Points (a), (b) and (c) should be repeated at five-yearly intervals. Workers who test negative for asbestos-related diseases and whose first exposure to asbestos is remote should not have to undergo further check-ups, but subjects should be informed that if specific symptoms occur they should consult their GPs who will, if necessary, refer them back to the programme. Subjects who test negative and whose first exposure was less remote should repeat the check-ups five years after joining the programme. Workers who test positive for asbestosis should be vaccinated yearly against influenza and pneumococcal disease, and be given five-yearly check-ups in accordance with the programme [63]. Medical examinations, LFT and CXR should in any case be suspended when reaching a predefined age. For further investigation (i.e., pulmonary nodules) properly equipped facilities should carry out further tests until a clear diagnosis is reached and treatment decided. Where the diagnosis and treatment of pleural mesothelioma are concerned, appropriate liaison will have to be arranged with facilities and personnel having the necessary experience in this field [64]. Smokers should be encouraged to give up smoking. All the instruments used must be consistent to requirements that, in the case of lung function tests (LFT) and chest X-ray (CXR) for occupational lung diseases, already exist [62,65,66,67,68,69].

Periodic reports on the programme should be forwarded to leading figures in the Local Health Units. The scientific community must be kept informed by means of publications. At intervals to be defined, the contents and calendar of the programme should be reviewed in the light of the characteristics of the participants and of scientific knowledge.

(4) *Fostering epidemiological research on asbestos*. Even if the law of asbestos ban did not specifically address the notion of fostering scientific research on asbestos, it indirectly favored the development of research that was supported by the National Research Council, the Ministry of Health and other authorities. The law established the National Conference of environmental and health safety of industrial technological processes, materials and products, was aimed at a wide range of participants, namely, experts, trade unions, companies, environmentalist and consumer associations, universities and research institutes. The First National Conference on asbestos was held in Rome in 1999, and provided the setting for presenting and discussing research findings, including epidemiological studies that threw light on previously insufficiently investigated items. The implementation of epidemiological surveillance of mortality for malignant pleural neoplasm in all 8000 Italian municipalities brought to light a number of high-risk areas such as the town of Broni in Lombardy where there was a large asbestos-cement plant [70] and the town of Biancavilla in Sicily that had a naturally occurring fluoro-edenite fibres in soil and building materials used in the local construction industry [71,72]. Both areas were subsequently included among National Priority Contaminated Sites.

Industries with significant asbestos presence that were object of epidemiological studies coordinated by Istituto Superiore di Sanità (ISS), partly published before the National Conference, partly presented there and published subsequently, included sea-transport [73], rock salt facilities [74], railway carriage construction and repair [75,76,77,78], asbestos-cement industry [79,80,81,82], chemical and warfare industry [83]. Researchers from ISS and other collaborating institutions also investigated high-risk populations including insulators [84], subjects compensated for asbestosis [85,86], and residents in areas with naturally occurring tremolite fibres [87,88].

Further support to epidemiological research was provided in the framework of the Second National Conference on asbestos held in Venice in 2012 [89], that triggered a two year research project including among else a major study on mesothelioma incidence in National Priority Contaminated Sites [90]. 

The three year research plan of INAIL (2016–2018) provides a specific area concerning asbestos including the harmonization of technical measurement of asbestos fibres, the surveillance and remediation of contaminated sites, the improvement of asbestos-related diseases epidemiological surveillance systems and the psychological support for asbestos related diseases victims and caregivers [91].

A public system for the insurance of workers against the risk of injuries and occupational diseases has been in place since about 1930, with the leading role of INAIL. Decree n. 626/94 significantly changed INAIL’s role, from an agency exclusively with insurance responsibilities to an agency with an active role and responsibilities in terms of planning, prevention, information, dissemination and support [55]. The merger of INAIL with the research institute ISPESL in 2010 was also part of this coordination strategy which entailed a greater role of INAIL for the research activities concerning health and safety at work. The prevention of asbestos-related diseases is a major topic in the INAIL research plan of action. Furthermore, the Institute provides technical and financial support to companies for asbestos removal and disposal.

(5) *Asbestos removal and waste disposal*. The 1992 ban has provided for the adoption of technical regulations to deal with both the general aspects of reclamation and particular aspects for which the reclamation works needed specific technical conditions [92]. Over the past 25 years, major reclamation projects have involved thousands of railway carriages, many military ships, chemical plants, and power plants. Many asbestos cement production sites have been converted to produce non-asbestos-containing fibrous cement and have also been reclaimed [93]. Today, the largest number of reclamations concerns the removal of industrial and civilian coverings made with asbestos cement and a much smaller amount for friable thermal insulations.

Data collected in some regions and from national institutions show that annual asbestos-removal work account for about 1% by weight of total asbestos-containing materials present in 1992, which means 75% are still to be recovered. The number of interventions has increased proportionally in the years in which economic incentives granted for the execution of reclamation. If no special economic conditions are created, the asbestos removal process will be completed by the end of the current century [89].

However, degradation of the material may occur over such a long time, because of weathering, and environmental conditions may be altered [94]. Asbestos cement reclamation operations do not present particular risks of fiber dispersion in the surrounding environment. On the contrary, the removal of crumbling materials is needed, above all in the industrial field and this requires sophisticated technical procedures to prevent the dust from becoming airborne [95].

Sealed boundary areas (static confinement) are placed under vacuum (dynamic confinement) to maintain the unavoidable pollution inside the confined area. The entrance and exit from these areas are through a tunnel. The exit is an especially critical point: staff must leave the clothes used for work in the first “dirty” dressing room, shower in the next compartment while still wearing their protective mask and finally access the dressing room where he wears his “street clothes” again. Bags containing the removed material also leave the work area in sturdy sealed plastic bags, which, after careful washing, are again sealed in new clean bags. Companies offering these services are registered in a public directory and are therefore authorized to carry out reclamations, because of their appropriate know-how [96]. The list of waste by type and quantity is described in a special form which is delivered to the manager, which is in turn authorized to transport hazardous wastes. The manager submits the form to the landfill at the time of delivery. In turn, the landfill returns the form to the waste producer, thus certifying that the required transfer procedure has been successfully completed. In Italy, landfills are listed by the type of materials they are able to dispose of. Waste containing asbestos is buried separately from other special wastes and covered with a few cubic meters of soil [97]. The location of these landfills is recorded in the municipal archives for future access. Choosing to bury asbestos appears to be the most reasonable and economical way, provided that sites are chosen from a suitable geological and logistic point of view. The geographic location of these sites should be equally distributed across the country to reduce energy costs for the travelling and the risk of road accidents.

(6) *Updating the asbestos removal workers protection legislation*. Italian legislation on dust protection in workplaces dates back to 1956 and was in force for asbestos until 1991 [98]. The law did not set limit values but simply stated that workers should be completely protected from dust exposure. The asbestos-related diseases that occur today are mainly the consequence of exposures that took place 30/50 years ago and therefore during the years in which the aforementioned law was in force. Clearly, the law was not properly applied. The final version of the legislation that protects the asbestos cleaners during work was introduced and enacted some 10 years ago [54]. A respiratory limit value, based on air quality standards for Western Europe published by the WHO in Copenhagen in 2000, has been introduced [99]. Hygienic accidents that may occur during work overcoming the respiratory limit value (0.01 ff/cc) must be recorded in a special register to be kept for at least 40 years.

According to Law 257/92 (art. 9) [12] companies that use asbestos “directly” and “indirectly” or carry out reclamation or disposal activities have a duty to send a yearly report to the Local Health Unit and the Regional Council. The content of the report includes the measures to be taken to protect the health of workers and the protection of the environment. For example, the Tuscany Region started to examine such activities in 1993, and has continued to do so to date. In addition to providing annual data on the quantities of asbestos removed, a list of workers working in the field of reclamation has also been established. The list is actually a cohort of workers who are potentially exposed to asbestos risk and nowadays totals nearly 6500 workers. Hours worked annually and job titles are also recorded. The latter enables us to estimate a level of possible exposure in semi-quantitative terms. Such companies are trained in working methods that refer to both individual and collective protection conditions. However, considering the material they work with, it seems very reasonable to frame them in a cohort that appears to be only exposed very slightly to asbestos, but at a higher level than that of the general population. It is therefore also an easily accessible population for the possible programming of health surveillance activities, health promotion activities and future epidemiological studies [100]. Art. 9 of Law 257/92 allows the public administration to obtaining useful information for the “asbestos elimination process” at all stages.

(7) *International cooperation in global public health perspective*. Italian scientific cooperation on asbestos has collaborated with countries still using asbestos to promote the prevention of asbestos-related diseases, sharing the vision of health equity in a global public health perspective [101,102]. This is consistent with the goal of contrasting health inequities causing the highest burden of preventable, unnecessary and unjust diseases affecting asbestos exposed communities in the countries currently using asbestos with respect to those countries that already banned asbestos and experienced prevention measures. On the basis of its standing experience within the context of a public health approach, Italy contributes by supporting national efforts of those countries for moving toward prevention and asbestos ban [103].

In this framework, ISS conceived the establishment of a scientific cooperation network involving institutions from Italy and Latin America because most of the countries in this region are still using asbestos, and some of them are also producers. In the past decade, the cooperation network has involved multidisciplinary skills for developing research, training and dissemination activities, thus merging experiences and skills of researchers from Italy, Brazil, Colombia, Bolivia and Ecuador [104,105,106]. Activities and results of this cooperation network have been endorsed by researchers participating in five Annual Conferences of the International Society for Environmental Epidemiology (ISEE) held in Ireland (2009), Spain (2011), USA (2014), Brazil (2015) and Italy (2016). Results obtained in Italy and Latin American countries corroborate the importance of developing international scientific cooperation in order to support national efforts for addressing the most critical asbestos issues in countries where asbestos continues to be used, in order to move towards prevention initiatives and asbestos ban. Sharing public health perspectives between cooperating partners is a key factor to ensure the effectiveness of the collaborative activities to be undertaken, according to the local/national priorities and the real needs of each country.

## 4. Key Factors for Asbestos Policy in Public Health Perspective

A roadmap for asbestos policy in a public health perspective has to include key factors playing a critical role for society at large.

*Involving public institutions and other relevant stakeholders.* The role of public institutions at different administrative levels has to be identified in order to assign specific responsibilities to progress towards the adoption and the implementation of asbestos policy in each country. A National Institute of Public Health may play a coordinating role for networking other institutions and involved authorities, as was the experience of ISS in Italy in the Eighties in the last century. It can take several steps and actions to promote and reinforce the process aimed to consolidate knowledge and reasons for asbestos banning in countries where asbestos use is still permitted. For example, gathering information about the occupational, environmental and public health effects of asbestos production and use, performing research studies on asbestos environmentally and/or occupationally exposed subjects, asbestos-related morbidity and mortality, identifying specific areas of high incidence of asbestos-related diseases, and transferring information to different local authorities about the asbestos hazard and effective prevention activities. A National Institute of Public Health of a country that has already banned the use of asbestos can also implement cooperation activity with academic and research institutions of countries that continue to use asbestos, and may act as a counsellor for establishing legislations to ban asbestos. Conferences, symposia, research papers, public statements are also important means to reinforce the entire process. All these steps are based on a strong relationships with national policy-making bodies, authorities, agencies.

In many countries, National Institutes of Occupational Safety and Health are independent agencies as opposed to National Institutions of Public Health. Cooperation between the two institutions is recommended. This was the case in Italy where ISS and ISPESL (the National Institute of Occupational Safety and Health), subsequently merged with INAIL (the Italian workers compensation Authority), worked together as previously explained.

In order to briefly examine the role played by stakeholders other than public institutions in the contrast to asbestos-related disease in Italy, a historical perspective is essential. Besides referring to the papers by Vigliani [23], Scansetti [107] and Carnevale & Chellini [108] as an introduction, it is well documented that trade unions were the main drivers of improvement in working conditions and compensation practices. In considering the leading role of workers’ organizations with respect to the adoption and enforcement of preventive actions in the workplace, the following points should be kept in mind. Since the post-World War II major industrialization in Italy (1950–1960), the labour movement developed a bargaining approach that was specific by industrial sector and firm. Even if health hazards were initially equated to monetary compensation, knowledge of the inherent links between industrial organization and health hazards was developed. Among others, this prompted workers’ surveys and research on working condition supported by the trade unions and by social scientists. In the late 1960s, the coming together of these multiple experiences led to the development of a system to identify and prevent health hazards [109]. As a consequence, the notion of ‘risk pay’ was abandoned and negotiations focussed in monitoring and improving working conditions [110]. This determined a response firstly by local authorities that established prevention services in the working environment after 1970, namely in industrialized districts with more progressive local governments. Subsequently, with the adoption of the 1978 Health Reform, a prevention service for the working environment was included in the 672 Local Health Authorities, which the country was divided in, covering about 60% in the following decade [111]. The public health response triggered by the labour movement’s request for prevention of health hazards in the workplace, was a favourable setting when the law banning asbestos was approved in 1992. At the time of adoption of the asbestos ban, most TUs were quite favourable, with some exceptions in those sectors that were going to face the largest job cuts such as asbestos mining and asbestos cement manufacturing. Following Law 257/92, and as previously discussed, the unions mainly focused on prevention of asbestos exposure for workers in asbestos removal and environmental clean-up in a wide range of settings. Concurrently, the struggle for equitable compensation practices for cases of asbestos-related diseases was also pursued.

In the meantime, the new stakeholders, the asbestos victims, their relatives and their associations offered a key contribution to the ongoing decision-making processes. The Associations of Victims of Asbestos and their Relatives (AFEVA) was established in Casale Monferrato (Piedmont Region) in 1988 and subsequently developed in many other parts of Italy where major asbestos industries had operated. Besides contributing to the ban of asbestos, AFEVA currently supports patients of asbestos-related disease with respect to medical care, social security, compensation and legal counselling in the courts. The role of victims’ associations has been investigated in series of anthropological studies by Agata Mazzeo [112,113,114]. The practices characterizing victims’ associations include care for the diseases and awareness of the importance of suffering, which is not only an intimate matter, but also the foundation of anti-asbestos activism aimed at the negotiation of rights and the promotion of change. This also requires exchanges with other social players, such as local politicians and elected officials, public health institutions, the judiciary and the transnational movement for the global asbestos ban. In this context, the priority was to give a voice to the sufferers and make the disasters caused by asbestos visible. The messages expressed by the victims must be shared with the scientific community (in order to be authoritative), present in the media, legitimized by the Law and, last but not least, integrated in the transnational scenario of activism. Remarkable consistency between Italian and Brazilian asbestos affected communities was brought to light by investigating Eternit victims’ associations operating in Casale Monferrato (Piedmont Region) and Osasco (São Paulo), respectively.

*Training*, *health literacy and communication on asbestos.* Specific training for asbestos exposed workers including those who are involved in the remediation or maintenance of the asbestos in place is needed in order to provide critical information on risks and health impacts caused by exposure to asbestos, and on the correct working methods and management of personal and collective protection devices. Sometimes the widespread presence of asbestos-containing materials and media information, which often tends to exaggerate health impacts, may generate a widespread fear in the population that in some cases may experience anxiety at the sight of an asbestos cement roof. The key point to focus on is disseminating the correct information to the population on the distinction between the concept of danger and hazard. It is right to inform on an asbestos danger as it is a widespread carcinogen throughout the country. It is not a hazard, though, if the fibres do not become airborne and are therefore possibly inhaled. Inhalation may happen whenever the material is mechanically disturbed or pulverized without adequate dust control [115]. The likelihood that fibres will be released is defined as a hazard. The hazard is therefore definable and quantifiable by analytical determination of the presence of airborne fibres. Unfortunately, this distinction is almost never respected by the media creating a misunderstanding as if danger= risk = impact on human health. The lack of health literacy in the affected population has negative consequences on the management of asbestos related problems.

Public protest often up-end public institutions’ intervention priorities, by forcing investment into reclamation work with a foreseeable zero impact on public health, rather than channelling the funds in activities that have beneficial effects on public health. Communication and dissemination of information on asbestos may be provided through public events as well as through mobile exhibitions on asbestos aimed at informing people about the important issues, for an effective approach on how to face the issue. An example of Italian mobile exhibition on asbestos namely “BastAmianto” (that is, StopAsbestos) was developed in Italy in 1991 aimed at popularizing and disseminating scientific information using language that could be understood by the general public. Knowledge is the basis for health literacy and the implementation of prevention [116]. The exhibition consists of information panels, old asbestos containing materials encased in plexiglas boxes, video and photographs. To date, this exhibition has been shown in numerous Italian cities and visited by many thousands of people. 

*Failures.* Notwithstanding the overall positive evaluation of the regulatory effort realized in Italy to ban the use of asbestos, it is useful to address some failures that have mainly to do with an excessive fragmentation of the tasks assigned to different public institutions dealing with environmental and health protection at a regional and local level. An example of this is reported in a recent paper on tremolite fibres contaminants of a feldspar mineral (1–2% by weight), extracted in Sardinia and distributed as raw material for the ceramic industry both in Italy and abroad [117]. This activity has been in existence for many years, implying occupational exposure to asbestos for thousands of unaware workers, and has not yet been definitively and safely terminated.

The above-mentioned fragmentation of regulations has also determined an overload of tasks for the members of the general public that, for instance, are supposed to self-assess quantity and location of asbestos-containing materials still in-place, in view of their removal. Obtaining this kind of information in a large proportion of cases and in a timely manner would require the introduction of tax rebates or at least financial incentives or bonuses.

## 5. Lesson Learned

Preliminary to listing a few most relevant “lessons learned”, this paper has focused on the actions that have determined the asbestos ban process both before and after the very adoption of the law itself. This mode of presenting the events, hopefully, might be beneficial for the readers, especially for those resident in countries where the use of asbestos in industry is still allowed.

- *Engaging local and central authorities avoiding delays between local and national decision-makers and fragmentation of regulations*. Banning the use of asbestos in a given country is necessarily a policy decision based on the awareness that the contribution of asbestos to economic development is fallacious, in as much as the economic costs of health care and environmental clean-up are not externalized, and that the distributive aspects of costs and benefits are duly considered. Furthermore, overwhelming scientific evidence clearly documents the dimensions of the health impact of asbestos including the future burden of disease associated with the long latency times of asbestos-related disease. Once such a decision is taken, consistency among the action of central and peripheral environmental health authorities has to be insured.
- *Training professionals and administrators. Engaging affected communities*. The pursuit of this aim requires a major health literacy campaign in order to develop a common language based on sharing accredited scientific evidence and evaluations. The process also requires training professionals, local authorities, and informing affected communities and the media.
- *Creation and implementation of national health and epidemiological systems of asbestos disease.* It is of primary importance to set up and implement a health information system concerning the occurrence of asbestos-related disease, including time and space coordinates and variation, as well as correlation with exposures circumstances. Concurrently, capacity building for asbestos fibre sampling and analysis must be performed. International scientific cooperation may ensure major support.
- *Policies for managing asbestos contaminated sites.* Given the widespread use of asbestos, the high costs of environmental clean-up (including appropriate procedures for waste management) and the unavoidable budgetary constraints, procedures for priority setting have to be put in place, considering the estimated number of preventable cases (taking into account the size and age structure of the exposed population, together with the current exposure levels), the feasibility aspects including the costs, and the equity implications of the decision-making process. The latter implies assigning priority to the worst-off settings, thus concentrating the efforts on marginalized and peripheral communities where environmental risks and unfavourable socioeconomic conditions may concurrently be operating.
- *Promoting further research on asbestos exposures and related health impacts where needed*. Finally, where needed, ad hoc research on previously unknown or underestimated exposure circumstances should be developed, in order to ensure a clear representation of the problem and to contribute to the advancement of knowledge that could be helpful even in different settings.

## 6. Conclusions

In 1992, the adoption of the law banning asbestos in Italy was a substantial achievement since Italy had been one of the main producers and consumers in 20th century in Europe. It was essential to strengthen a set of actions in order to advance in the exit process (still on-going) from the industrial model requiring the use of asbestos. These actions primarily concern public policies for asbestos substitutes, national surveillance system of mesothelioma incidence, health surveillance for asbestos ex-exposed subjects and asbestos exposed workers essentially in remediation works, asbestos removal and waste disposal as well as epidemiological research on asbestos.

Based on the Italian experience, a roadmap for asbestos policy in a public health perspective has to include key factors playing a critical role for society, such as the involvement of public institutions and other relevant stakeholders, training plans, health literacy and communication on asbestos.

Notwithstanding the overall positive evaluation of the regulatory effort in Italy dedicated to ban the use of asbestos, this paper illustrates and discusses the failures of an excessive fragmentation of the tasks assigned to different Italian public institutions dealing with environmental and health protection at a regional and local level in order to highlight potential risks.

Key factors and lessons learned in Italy from both successful and ineffective asbestos policies are here described to support the relevant stakeholders in countries still using asbestos with the aim of contributing to the termination of asbestos use. In particular, this paper has pointed out the relevance of: (i) engaging local and central authorities avoiding both delays between local and national decision-making and fragmentation of regulations; (ii) training professionals and administrators; (iii) engaging affected communities; (iv) implementing policies for managing “asbestos contaminated sites;” (v) promoting further research on asbestos exposures and related health impacts especially for previously unrecognised or underestimated risks.

Finally, the relevance of developing international scientific cooperation on asbestos between countries that have and have not banned asbestos has been discussed emphasizing the need to share the vision of health equity in a global public health perspective.
